# Study of dielectric modulated dual source triple gate TFET for biosensing applications

**DOI:** 10.1186/s11671-025-04395-4

**Published:** 2025-11-24

**Authors:** Guowei Cui, Huifang Xu

**Affiliations:** Institute of Electrical and Electronic Engineering, Anhui Science and Technology University, Fengyang, 233100 Anhui China

**Keywords:** Dual-source, Triple-gate, Biosensor, Sensitivity

## Abstract

A dielectric modulated dual-source triple-gate tunnel field effect transistor (DM-DSTG TFET) based biosensor is proposed for the detection of biomolecules, the performance of the proposed biosensor was rigorously evaluated using the Silvaco Atlas simulator. The dual-source is positioned within the two voids of the triple parallel gates, while cavities located between the source, a portion of the channel and the gate are specifically designed for biomolecule conjugation. Variations in the device's electrostatics, including the electric field, surface potential, and energy band diagrams, are analyzed in response to changes in the dielectric constant of different biomolecules, thereby reflecting the biorecognition process within the biosensor. The results demonstrate that the proposed device exhibits enhanced sensitivity (10^10^) and a reduced subthreshold swing (32.7 mV/decade), highlighting its superior performance. The feasibility of the biosensor as a label-free detection platform is further validated using avian influenza virus and DNA as target biomolecules. Consequently, the DM-DSTG TFET based biosensor emerges as a highly promising candidate, offering advanced detection and recognition capabilities for future biosensing applications.

## Introduction

Over the past few decades, biomolecules have been recognized as pivotal elements in pharmaceutical science, particularly in applications such as disease detection, where their role is indispensable for advancements in cell engineering and the exploration of biological mechanisms [1–3]. In tunnel field effect transistor (TFET) devices, the gate oxidation region is fabricated as a cavity that can be used to hold biomolecules. Since the interaction between biomolecules and transduction elements can affect the electrical properties of biosensor, it is possible to translate the recognition of biomolecules into the detection of electrical signals of devices [4, 5]. Accurate and rapid detection of disease markers is essential to any diagnostic process. Early detection of the disease can improve the cure rate and reduce unnecessary losses [6, 7]. The traditional testing process requires professional technicians, high cost, and large space requirements. In contrast, the TFET based biosensor has the characteristics of low cost, easy to use and highly sensitive in the detection of disease markers [8]. At the same time, compared with metal oxide semiconductor field effect transistor (MOSFET), TFET has the advantage of reducing the short channel effect and subthreshold swing ($SS$) due to its band to band tunneling mechanism [9–11].

The conventional TFET (C-TFET) is characterized by a P-I-N structure, where the source and drain are heavily doped, while the channel remains intrinsically doped. However, due to inherent limitations such as random doping fluctuations and the dominance of simple point tunneling mechanisms, the drain current of the C-TFET is often constrained and may not achieve the anticipated levels of performance [12, 13]. In many studies, researchers have tried to explore different techniques to improve sensing capabilities, such as the dielectric modulated double source trench gate TFET based biosensor was proposed by Chen et al. [14] for the detection of biomolecules, and has shown promising application prospects. In 2016, Goswami et al. [15] proposed a circular gate TFET, although the device can reduce the ambipolar current and improve the on-current and off-current ratio ($I_{on} /I_{off}$), it is difficult to realize the circular gate in the process. The C-TFET has a lower drain current, so many researchers have made many improvements in the gate structure to improve the drain current. For example, surrounding-gate [16, 17] and double-gate [18] biosensors have certain advantages, but none of these structures are as easy to fill with biomolecules than trench gate structure [19–21]. A dielectric modulated 4H-SiC source triple metal gate-all-around silicon carbide field effect transistor (FET) biosensor with dual-sided cavities was proposed by Yadav et al. [22] for label-free detection of biomolecules such as gelatin and DNA, exhibiting significantly enhanced sensitivity over silicon-based devices. A triple metal gate electrostatically doped vertical nanowire TFET was proposed by Bhardwaj et al. [23], which demonstrates superior performance in parameters such as on-state current ($I_{on}$), $I_{on} /I_{off}$, and transconductance compared to single-gate and double-gate structures. In this study, a vertical structure is employed, where the incorporation of triple-gate significantly enhances the gate control over the current. The central vertical gate, resembling a trench gate configuration, is particularly advantageous for the placement of biomolecules, thereby optimizing the device's functionality for biosensing applications. Therefore, a novel dielectric modulated dual-source triple-gate TFET (DM-DSTG TFET), which employs dual-source and triple-gate is proposed to obtain an ultra-high drain current sensitivity ($S_{{I_{on} }}$).

In this paper, the performances of DM-DSTG TFET based biosensor in terms of $S_{{I_{on} }}$ are discussed in depth. Section 2 outlines the device structure and simulation model utilized in this study, while Sect. 3 delves into the influence of various factors on the electrical properties of the DM-DSTG TFET based biosensor, including the electric field, surface potential, and energy band. Special emphasis is placed on the distinct impacts of different biomolecules and charged biomolecules on the transfer characteristics. Avian influenza virus and DNA were used as target molecules and the selectivity of the device was briefly analyzed. Section 4 summarizes the research results and draws conclusions.

## Device structure and simulation model

### Device structure

**Fig. 1:**
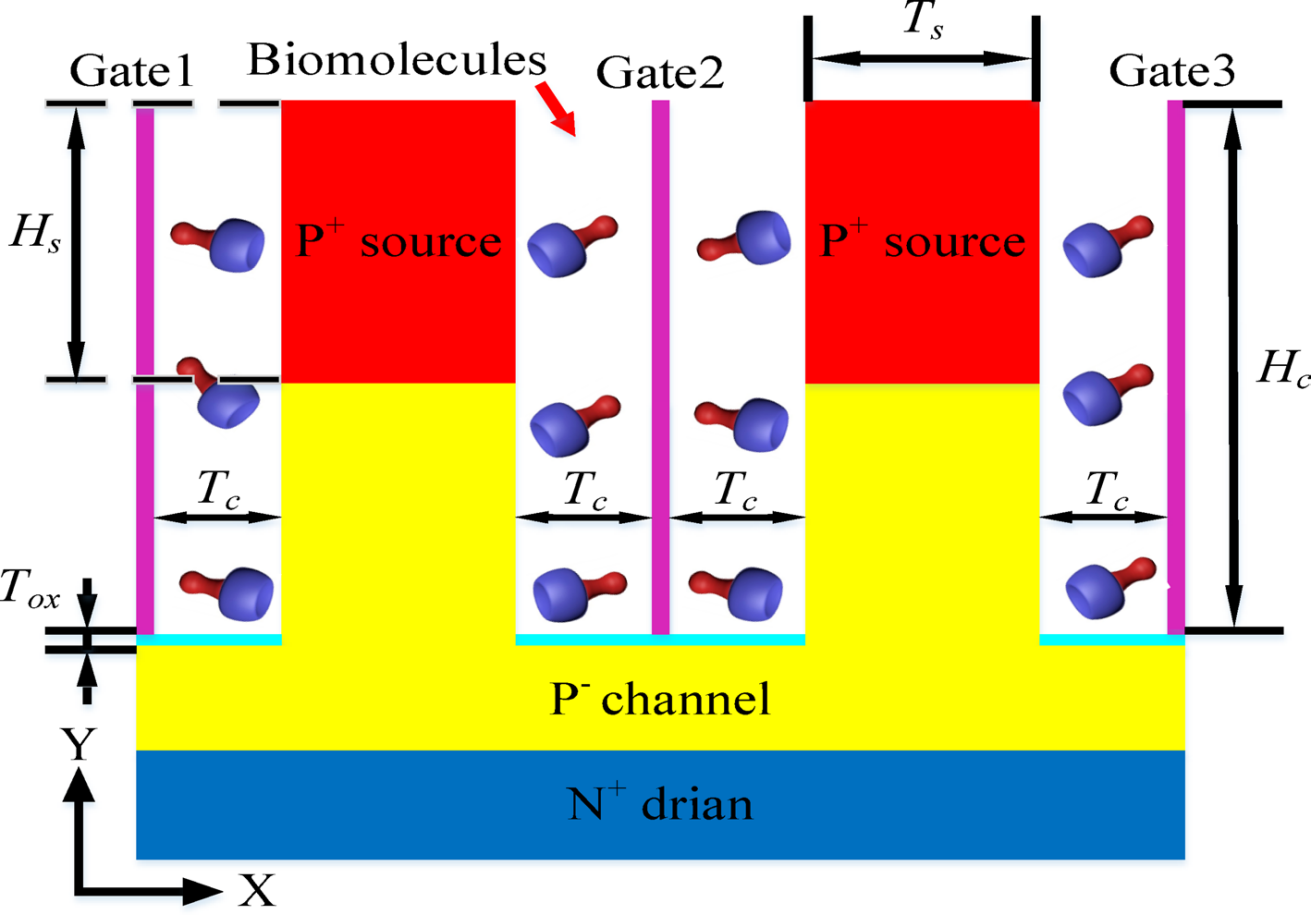
Schematic of DM-DSTG TFET based biosensor

The schematic of DM-DSTG TFET based biosensor is shown in Fig. 1. In the figure, it can be seen that dual-source and channel are located in the middle region of the gate. Four cavities are located on both sides of the source and channel. Although the 5 nm cavity is smaller than certain target biomolecules, their critical epitopes can partially penetrate into the cavity, inducing detectable signal changes through the epitope penetration mechanism where only specific functional regions of large biomolecules enter the nanoscale cavity [24]. An oxide layer of silicon dioxide with a thickness of 1 nm is added to act as an insulating layer, isolating the gate and preventing current leakage. Triple-gate with work functions of 4.7 eV was deposited on one side of the cavity in order to improve the sensitivity. The source and channel are P-type doping, and the drain is N-type doping. The structural parameters related to device simulation are detailed in Table 1. Compared to conventional dual-gate or trench-gate structures, the dual-source triple-gate structure proposed in this study incorporates additional gate and source, effectively forming two parallel dual-gate that create dual carrier tunneling paths. Simultaneously, it increases the number of cavities, improving space utilization. The two outer gates in the new structure further enhance the influence of the cavities on device characteristics and strengthen band bending at the tunnel junction, thereby promoting tunneling probability.

**Table 1:** Parameters value of DM-DSTG TFET based biosensor

Parameters	Symbol	value
Cavity width	T_C_	5 nm
Source width	T_S_	10 nm
Oxide layer width	T_OX_	1 nm
Cavity height	H_C_	29 nm
Source height	H_S_	15 nm
Source doping	N_S_	2 × 10^19^ cm^−3^
Channel doping	N_C_	1 × 10^16^ cm^−3^
Drain doping	N_D_	1 × 10^18^ cm^−3^
Work function	WF	4.7 eV

**Fig. 2:**
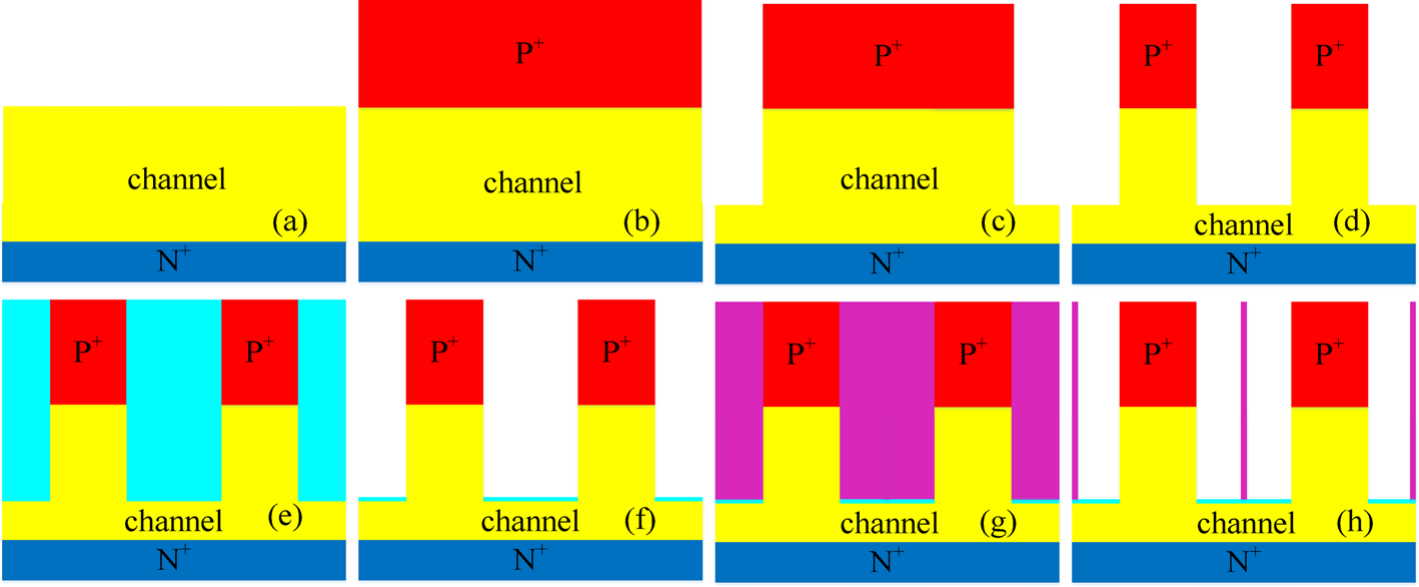
Fabrication flow for DM-DSTG TFET based biosensor

The fabrication process of the DM-DSTG TFET based biosensor is similar to the structure reported in Ref. [25]. Figure 2 illustrates the process flow of the DM-DSTG TFET based biosensor. First, as shown in Fig. 2a-d, the lightly doped silicon substrate undergoes masking, exposure, etching, ion implantation, and annealing to form the drain at the bottom of the device. For the source, a silicon-based source is grown via chemical vapor deposition to achieve heavy p-type doping. The corners above the source and channel are etched away, and the middle section is subsequently etched to form a trench gate. Next, as depicted in Fig. 2e-h, SiO₂ is deposited into the etched regions to form a 1 nm-thick oxide layer. Through metallization, a metal layer is deposited over the gate oxide to serve as the gate electrode. Finally, part of the gate is etched away to create space for biomolecules.

### Simulation model

**Fig. 3:**
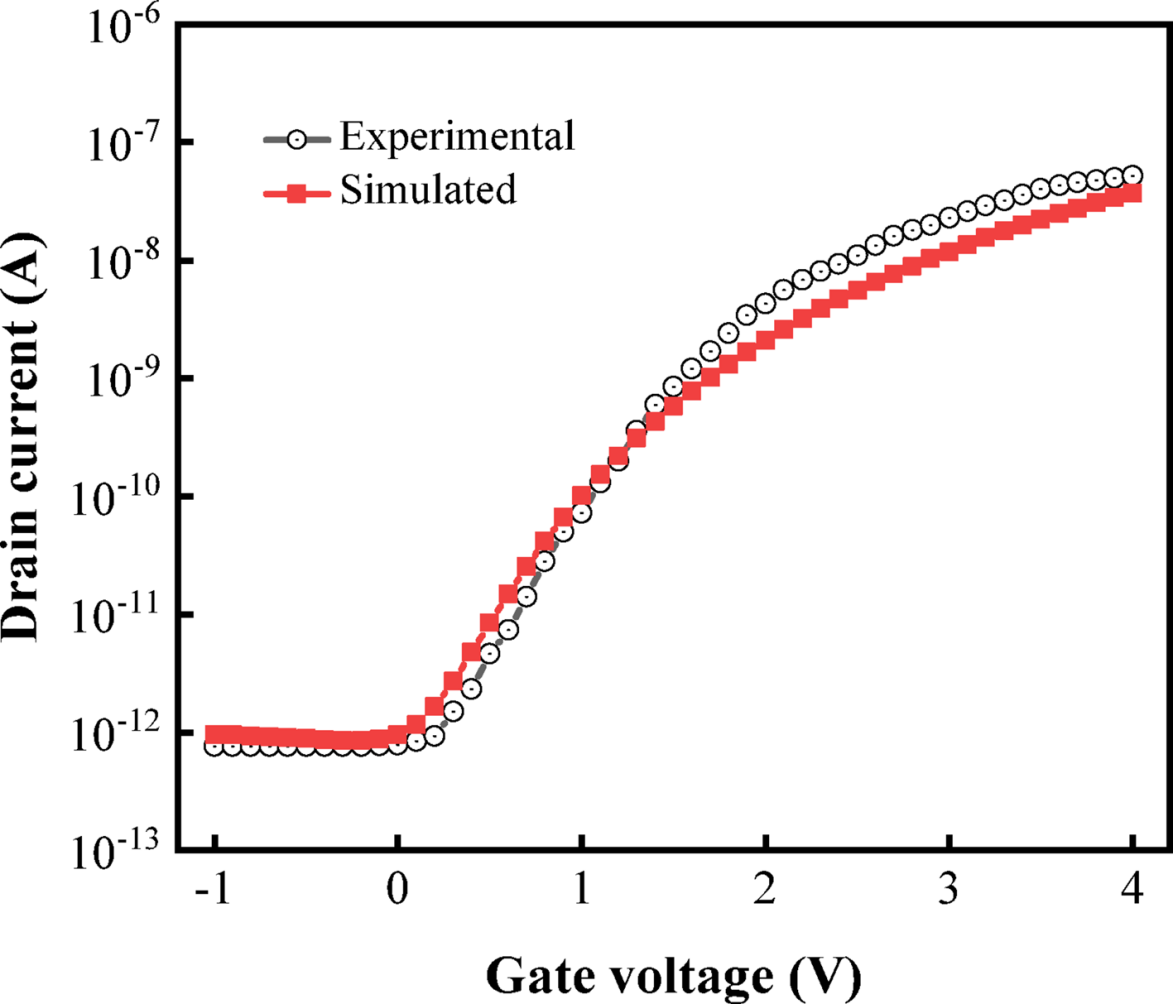
Comparison between simulation results and published experimental data

In order to deeply explore the performance characteristics of DM-DSTG TFET based biosensor, Silvaco Atlas TCAD tool [26] was used to simulate and analyze its sensitivity. It is well-established that the key to ensuring simulation accuracy is to choose a suitable model. Therefore, a standard band-to-band tunneling model is employed to describe the spatial variations of the energy band structure. The standard BBT model offers computational efficiency by means of analytical tunneling approximations while retaining acceptable accuracy for conventional device simulations. Its parameterized formulation enables straightforward integration with classical transport frameworks. In addition, Shockley–Read–Hall (SRH) reconstructions, Fermi–Dirac statistics, Lombardi mobility, Auger recombination model, and bandgap narrowing model were also used in the simulation. The SRH model is utilized to simulate thermally generated leakage currents, Fermi–Dirac statistics are invoked to account for property variations in highly doped regions, and the Auger recombination model is employed to describe direct recombination processes under high carrier concentrations [27–29]. The simulation employs a Gummel-Newton hybrid iteration method to solve the coupled partial differential equations in semiconductor device modeling, effectively balancing convergence efficiency and numerical stability. This study calibrated the NTFET based structure proposed by Wang et al. [30] through optimization of the parameters in the tunneling model. Figure 3 demonstrates excellent consistency between our simulation results and the experimental transfer characteristics.

Subthreshold swing sensitivity ($S_{SS}$), and $S_{{I_{on} }}$ are analyzed in detail [31, 32], and they are written as

1 $$ S_{SS} = \frac{{SS_{air} - SS_{bio} }}{{SS_{air} }} $$

2 $$ S_{{I_{on} }} = \frac{{I_{on(bio)} - I_{on(air)} }}{{I_{on(air)} }} $$

where $SS_{air}$ and $I_{on(air)}$ are the subthreshold swing and on-state current, respectively, when the cavities are filled with air. Similarly, $SS_{bio}$ and $I_{on(bio)}$ are the subthreshold swing and on-state current, respectively, when the cavities are filled with biomolecules.

## Results and discussion

### The influence of different biomolecules on biosensor

**Fig. 4:**
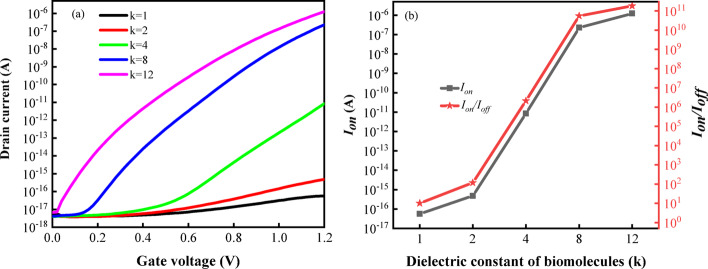
**a** Transfer characteristics, **b** $I_{on}$ and $I_{on} /I_{off}$ of DM-DSTG TFET based biosensor for different dielectric constant of biomolecules at $V_{ds}$ = 0.55 V and $V_{gs}$ = 1.2 V

Various biomolecules have different dielectric constants (k). For example, the dielectric constants of Protein A and Apomyoglobin are 4 and 8 [33], respectively. Differences in the dielectric constants of these biomolecules affect the capacitance value of the cavity, which in turn affects the drain current. Therefore, the detection of biomolecules can be achieved by monitoring the change in current [34, 35]. In this study, an air environment with a dielectric constant of k = 1 was selected to simulate the absence of biomolecules. As shown in Fig. 4a, the transfer characteristic curve of the device shifts to the left as the dielectric constant rises. It is worth noting that with the increase of the dielectric constant of the biomolecule, the drain current increases simultaneously when the drain-source voltage ($V_{ds}$) is 0.55 V and gate-source voltage ($V_{gs}$) is 1.2 V. The highest $I_{on}$ reaches 10^–6^ A, while the off-state current ($I_{off}$) is only 10^–18^ A at k = 12 in this structure. Figure 4b shows the influence of the dielectric constant of the biomolecules on $I_{on}$ and $I_{on} /I_{off}$ for the DM-DSTG TFET based biosensor. As the dielectric constant increases, the band bending becomes more severe, resulting in a smaller potential barrier width at the source-channel junction, thereby enhancing the probability of tunneling and increasing $I_{on}$ and $I_{on} /I_{off}$.

**Fig. 5:**
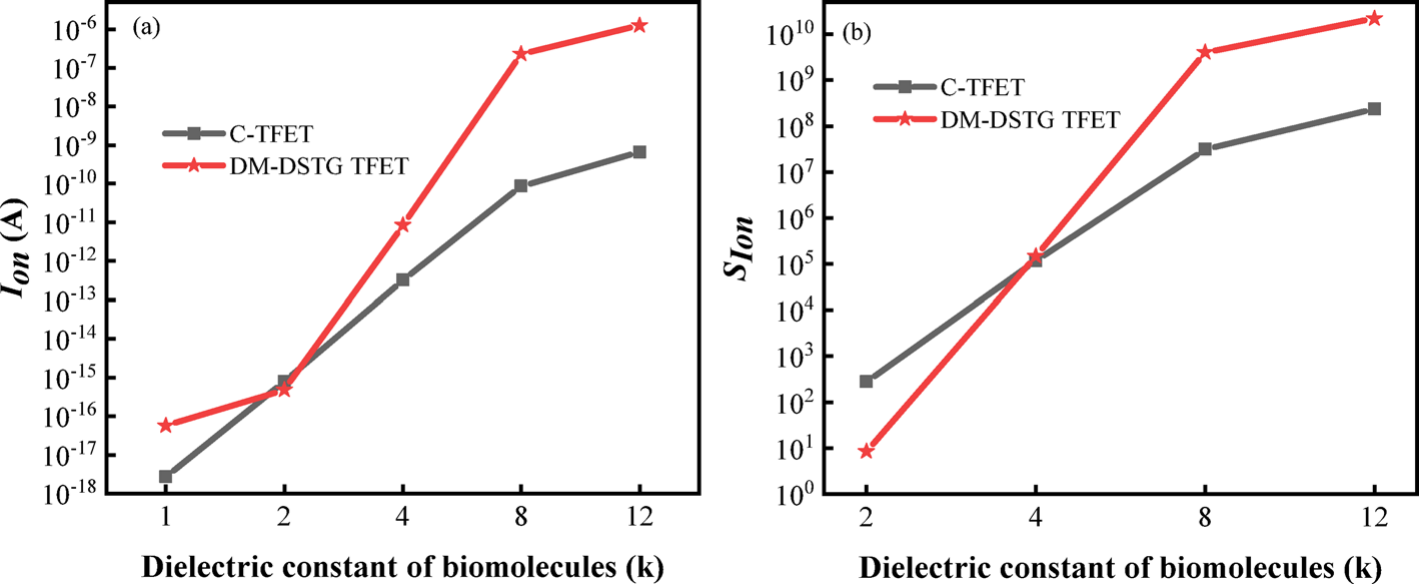
**a** $I_{on}$_,_**b** $S_{{I_{on} }}$ of DM-DSTG TFET and C-TFET based biosensor for different dielectric constant of biomolecules

Figure 5 illustrates the influence of the dielectric constant of various biomolecules on the performance of DM-DSTG TFET and C-TFET based biosensor. As depicted in Fig. 5a, the variation range of $I_{on}$ for the DM-DSTG TFET based biosensor is significantly broader than that of the C-TFET based biosensor, indicating a superior ability to distinguish between different biomolecules. Linearity is a critical metric for quantifying the responsiveness of a sensor to variations in input parameters. To validate the linearity of the proposed biosensor, this study analyzed the Pearson correlation coefficient between the logarithm of $I_{on}$ and the dielectric constant of biomolecules [36]. The degree of fitness (r^2^) was calculated to be 0.95, indicating a strong positive linear correlation between the dielectric constant and $I_{on}$. Figure 5b demonstrates that $S_{{I_{on} }}$ for the DM-DSTG TFET based biosensor increases from 10^0^ to 10^10^ as the dielectric constant rises from 2 to 12. This enhancement is attributed to the strengthened electric field near the tunnel junction, which reduces the tunneling width and consequently increases $S_{{I_{on} }}$. Notably, the maximum $S_{{I_{on} }}$ achieved by the DM-DSTG TFET based biosensor is 10^10^, whereas the C-TFET based biosensor reaches only 10^8^. A higher $S_{{I_{on} }}$ response signifies improved sensing speed and capability, underscoring the exceptional sensing performance of the DM-DSTG TFET based biosensor.

**Fig. 6:**
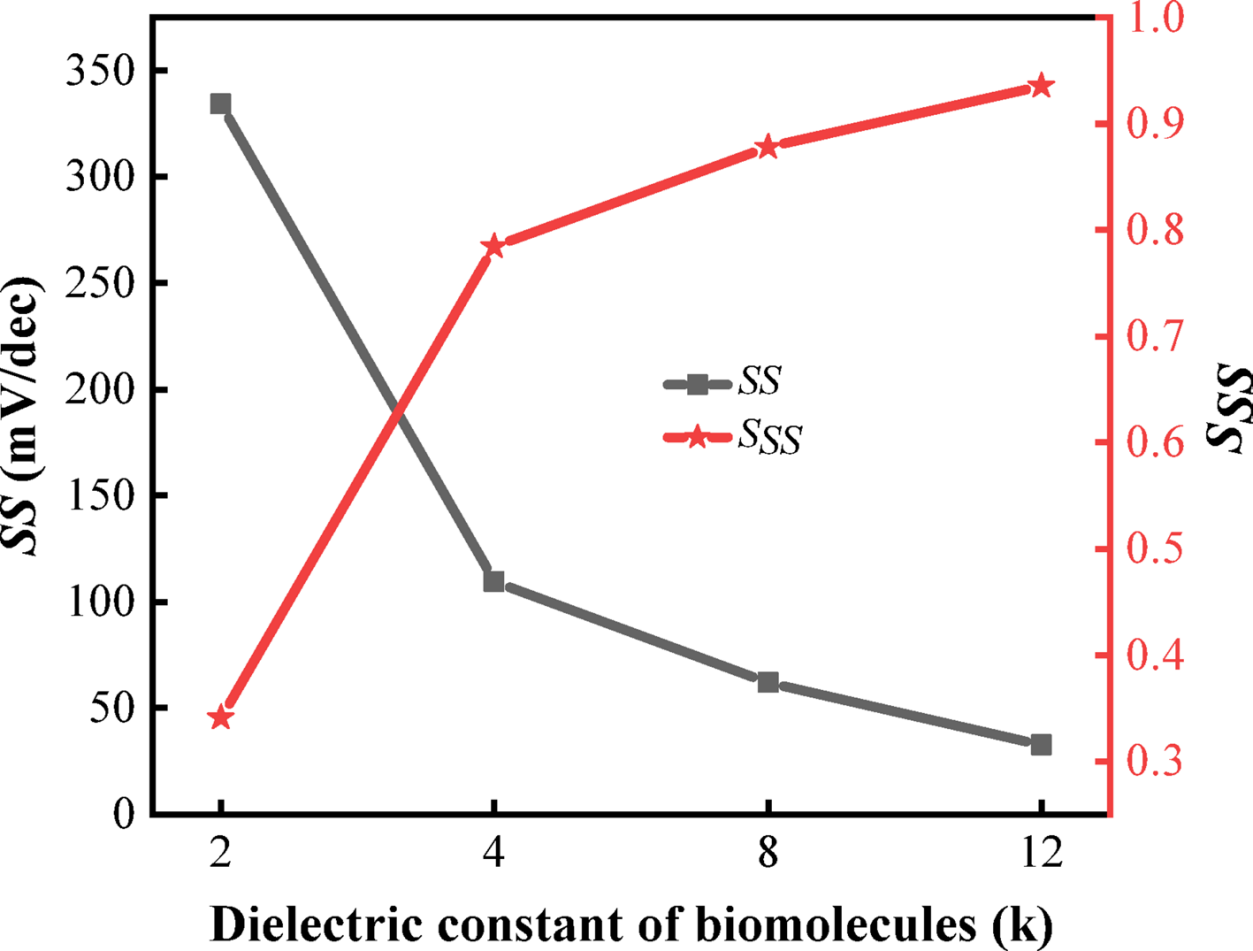
$SS$ and $S_{SS}$ of DM-DSTG TFET based biosensor for different dielectric constant of biomolecules

Figure 6 illustrates the variation of $SS$ and $S_{SS}$, where $SS$ is observed to decrease while $S_{SS}$ increases due to the higher dielectric constant of the biomolecule within the cavity. As depicted in the figure, the minimum $SS$ achieved is 32.7 mV/decade, indicating an enhanced gate voltage sensitivity in the subthreshold region. A smaller $SS$ signifies that the transistor exhibits greater responsiveness to gate voltage changes, which is attributed to the reduced tunneling barrier and improved carrier transport efficiency under higher dielectric conditions. Furthermore, the reduced $SS$ value enables the achievement of equivalent $I_{on}$ at significantly lower operating voltages. This characteristic to some extent reflects the device's enhanced resilience against aging and hot carrier effects [37, 38]. The maximum value of $S_{SS}$ is determined to be 0.93, which further substantiates the reliability of $SS$ as a parameter for distinguishing between different biomolecules in the device. This result not only enhances the device's sensitivity to biomolecular interactions but also contributes to lower power consumption during switching operations, making it highly suitable for low-power biosensing applications. Compared to the current sensitivity shown in Fig. 5b, the $S_{SS}$ reflects subtle changes in gate control efficiency, while the $S_{{I_{on} }}$ captures the overall response strength induced by biomolecular binding. When considered together, they evaluate the sensor's performance from complementary perspectives.

**Fig. 7:**
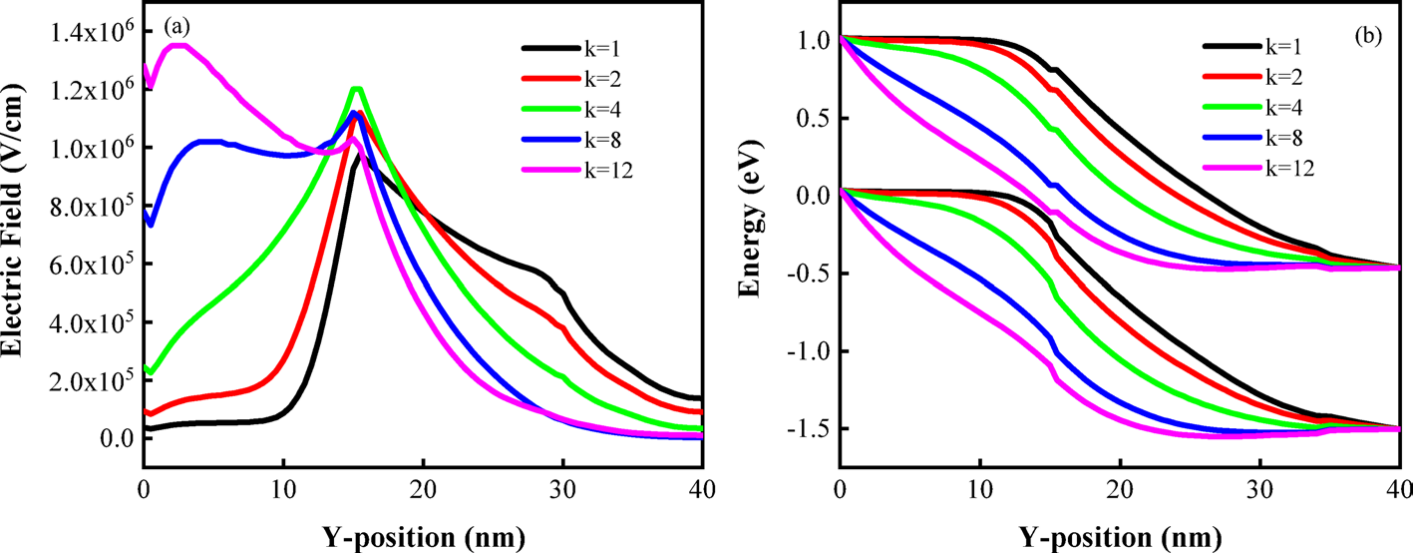
**a** Electric field, **b** energy band diagrams of DM-DSTG TFET based biosensor for different biomolecules

Figure 7 shows various electrostatic parameters such as electric field and energy band in the presence of air and biomolecules in the cavity when the $V_{gs}$ is 1.2 V. It can be clearly seen from Fig. 7a that the electric field in the source decreases with the decrease of the dielectric constant of the biomolecule. For higher value of k, this results in tighter electric field lines, which produces a higher electric field at the same voltage. At the same time, there is a peak in the electric field near the tunneling junction. This high electric field helps electrons tunnel from one energy band to another. Figure 7b presents the band diagram of the sensor, which is utilized to determine the tunneling width, defined as the minimum distance between the valence band of the source and the valence band of the channel. It is observed that the effective tunneling width near the source-channel junction progressively decreases as the dielectric constant of the introduced biomolecules increases.

**Fig. 8:**
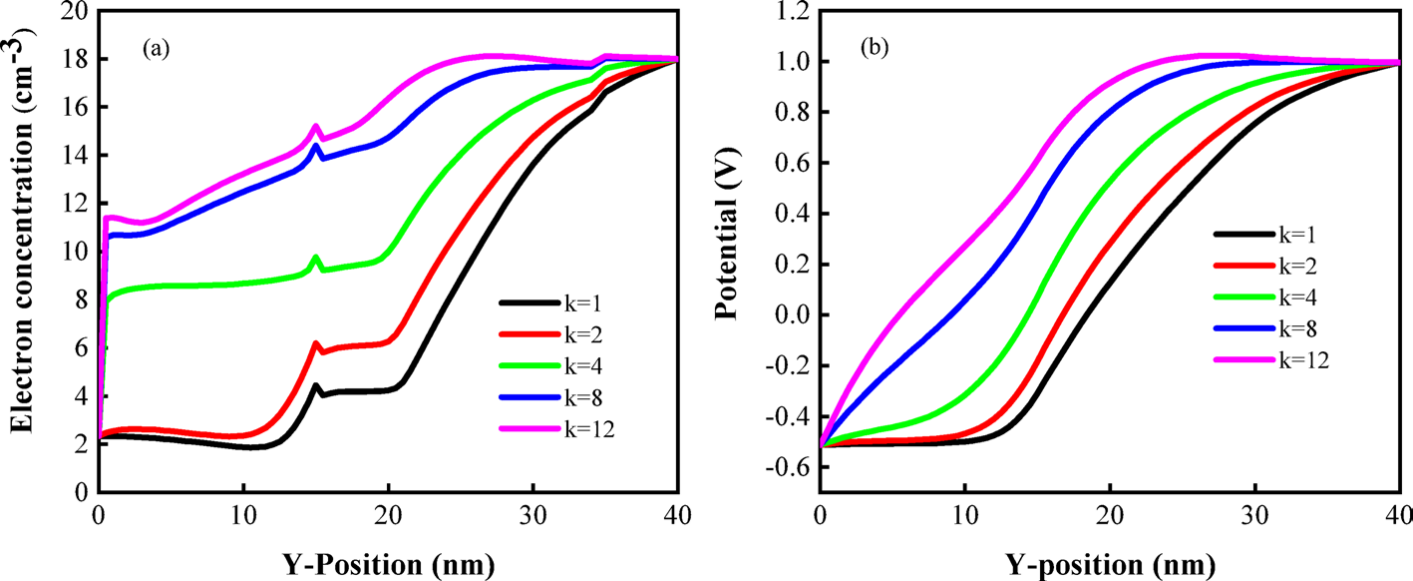
**a** Electron concentration, **b** surface potential diagrams of DM-DSTG TFET based biosensor for different biomolecules

Figure 8a shows the distribution of electron concentration along the Y-position. Due to the change in the dielectric constant, there is a significant difference in band bending between the two sides, which ultimately leads to an increase in electron concentration. Surface potential distribution is shown in Fig. 8b. Materials with high dielectric constant can accommodate stronger electric fields, thus increasing the electric field strength in the cavity, which in turn elevates the surface potential. Therefore, it can be seen from the figure that there are different surface potential curves, and among all biomolecules, the surface potential reaches the minimum when air is present. Thus, the detection effect of the sensor is remarkable.

### Impact of charged biomolecules on DM-DSTG TFET based biosensor

**Fig. 9:**
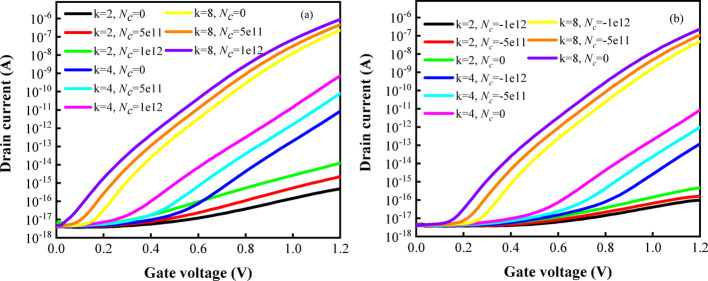
Transfer characteristics of DM-DSTG TFET based biosensor with dielectric constant (k = 2, 4, and 8) for **a** positive, **b** negative charge densities

In Fig. 9a, biomolecules with varying positive charge densities ($N_{c}$) are immobilized within the cavity, accompanied by dielectric constants of 2, 4, and 8. The introduction of positive charges into the cavity generates a vertical electric field directed toward the channel. This field causes the energy bands to bend downward near the source-channel junction, thereby reducing both the width and height of the tunneling barrier. Such modulation significantly enhances the band-to-band tunneling probability, leading to a notable increase in $I_{on}$. Conversely, as depicted in Fig. 9b, negative charges in the cavity produce a vertical electric field directed away from the channel, resulting in upward band bending. This increases both the width and height of the tunneling barrier, thereby suppressing the tunneling process and reducing $I_{on}$. Furthermore, it is evident that the transfer curves for both positively and negatively charged biomolecules span a wide range under different biomolecules.

**Fig. 10:**
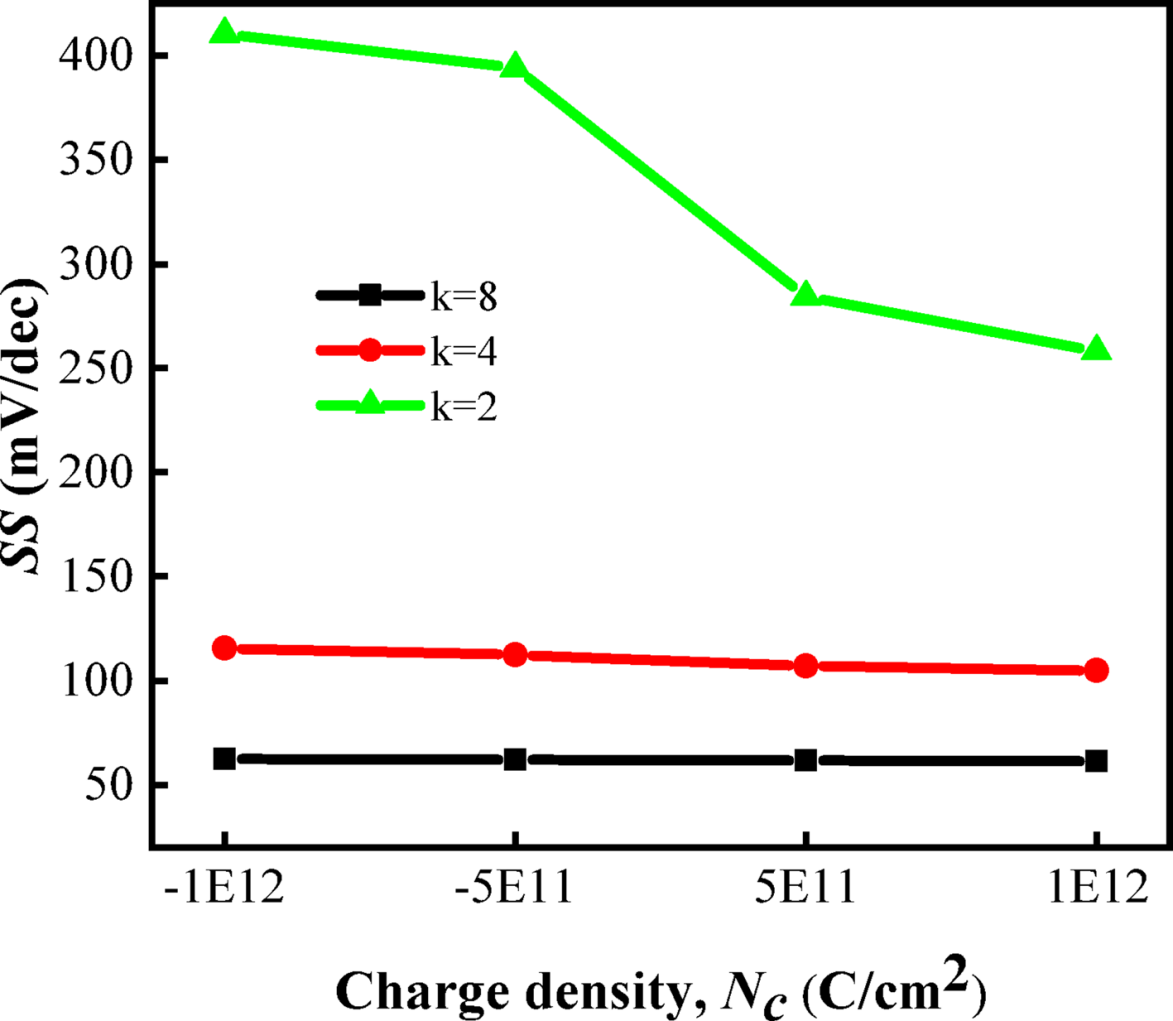
$SS$ of DM-DSTG TFET based biosensor for different $N_{c}$ at different biomolecules

The variation of $SS$ for different charged biomolecules is also illustrated in Fig. 10 to verify the sensing characteristics in response to the variation. As can be seen from the figure, $SS$ decreases with the decrease of dielectric constant, and $SS$ reaches the minimum value when k = 2. It can be seen from the abscissa that $SS$ decreases with the increase of $N_{c}$, and $SS$ has a small change trend but generally decreases when k = 4 and 8.

### Application of DM-DSTG TFET based biosensor in detecting Avian influenza virus and DNA

**Fig. 11:**
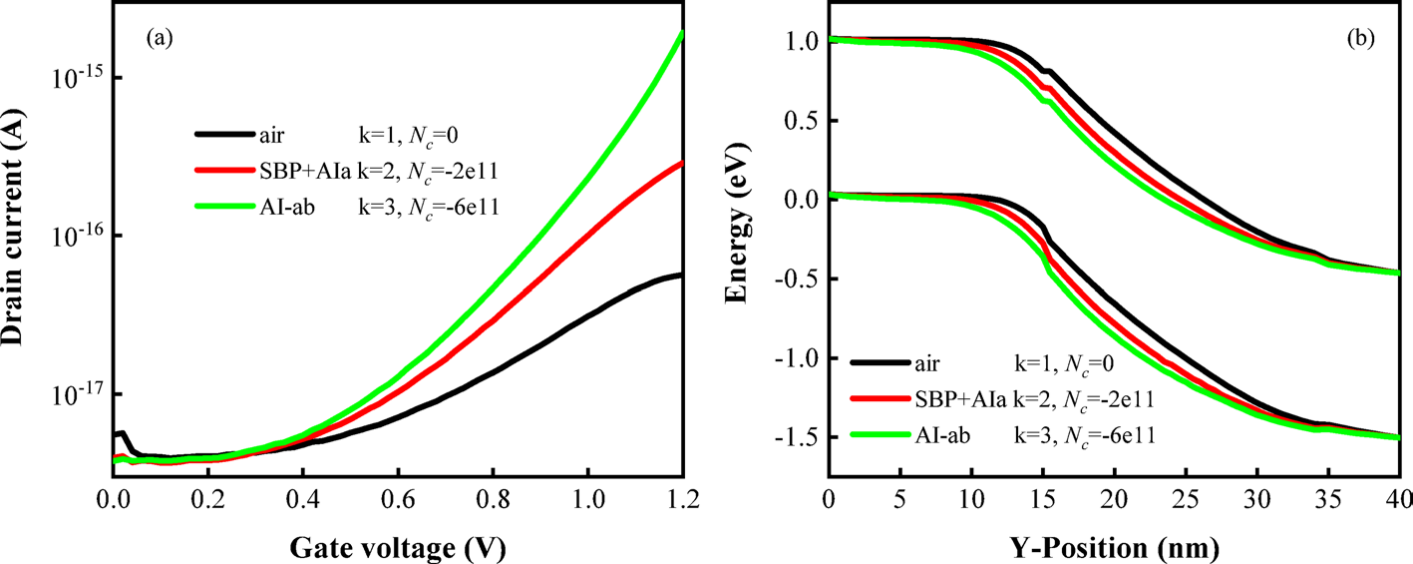
**a** Transfer characteristics, **b** energy band diagrams of DM-DSTG TFET based biosensor for SBP + AIa and AI-ab

The main biomolecules analyzed in the study were avian influenza virus and DNA. These molecules, enzymes, or biochemical substances are employed as suitable bioreceptors due to their ability to specifically bind to target analytes, thereby enhancing the detection capabilities of the sensor. Avian influenza antigens (AIa), which are specific proteins or molecular components located on the surface of avian influenza viruses, are recognized by the host's immune system, triggering the production of antibodies. Therefore, the AIa can be effectively detected by immobilization of Silica-binding protein (SBP) in the cavity [39, 40]. The charged biomolecules studied include double-stranded DNA (ssDNA), single-stranded DNA (dsDNA), AIa, and avian influenza antibody (AI-ab). The detection of AIa is combined with SBP, its effective dielectric value k = 2, surface charge density $N_{c}$ =  − 2 × 10^11^ C/cm^2^, the combination of AI-ab with the basal layer is expressed by k = 3 and $N_{c}$ =  − 6 × 10^11^ C/cm^2^. A ssDNA probe layer is pre-constructed on the nanoscale cavity surface for DNA detection, establishing the device's selectivity toward the target DNA. ssDNA is simulated with k = 2, surface charge density $N_{c}$ =  − 1 × 10^11^ C/cm^2^, dsDNA is simulated with k = 8 and $N_{c}$ =  − 5 × 10^11^ C/cm^2^ [41–43]. In Fig. 11a, drain current is plotted according to $V_{gs}$, drain current increasing order: air, SBP + AIa, and AI-ab. Due to the elevated dielectric constant of AI-ab, the highest drain current is achieved. In order to reflect the device's distinction between antigens and antibodies, selectivity ($\Delta S_{{I_{on} }}$) is often used to distinguish different types of biomolecules, which is expressed as the relative ratio of drain current between two different biomolecules [44], as shown in Formula (3).

3 $$ \Delta S_{{I_{on} }} = \frac{{I_{on(bio2)} - I_{on(bio1)} }}{{I_{on(bio1)} }} $$

where $I_{on(bio2)}$ and $I_{on(bio1)}$ are on-state current of two different biomolecules, respectively. By calculation, the $\Delta S_{{I_{on} }}$ of the device to avian influenza antigen and antibody is 5.62. Figure 11b shows the energy band diagram of DM-DSTG TFET based biosensor. With the increase of dielectric constant, the energy band near the device tunneling junction bends significantly due to the increase of capacitance effect. Variations in energy band can also be utilized as characteristic parameters of the sensor to differentiate between distinct biomolecules.

**Fig. 12:**
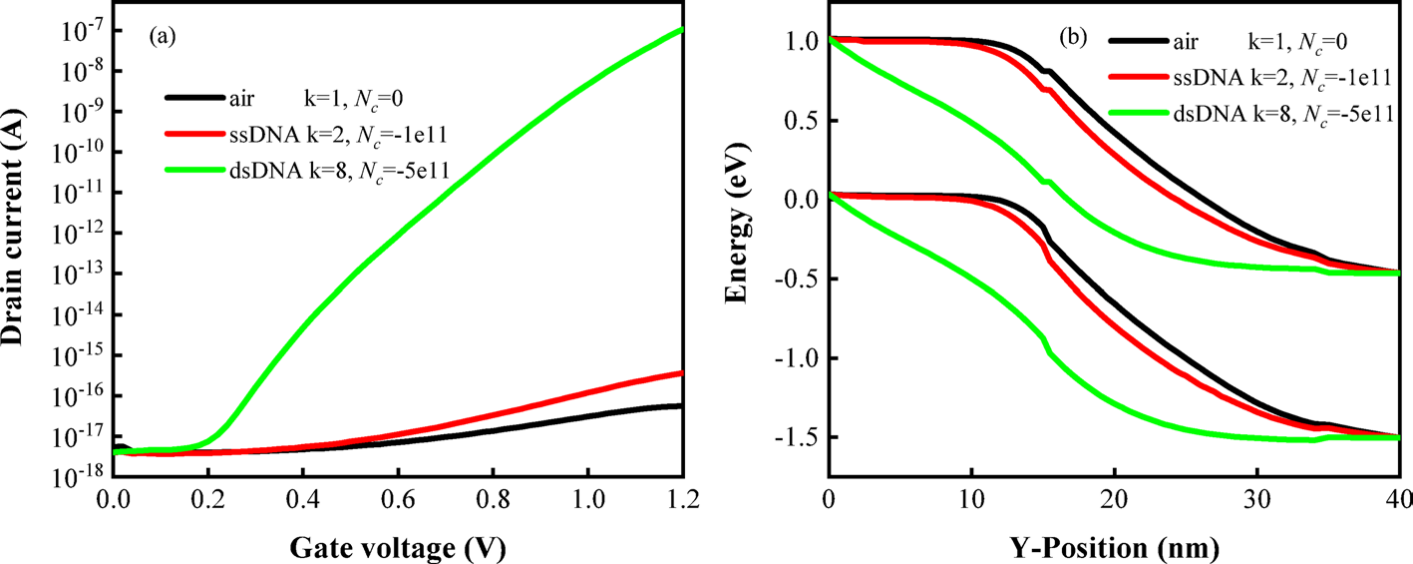
**a** Transfer characteristics, **b** energy band diagrams of DM-DSTG TFET based biosensor for dsDNA and ssDNA

As shown in Fig. 12a, ssDNA and dsDNA have different drain currents. It is worth noting that with the increase of the dielectric constant of the biomolecule, the drain current increases. According to the calculation of $I_{on}$ in the figure, it can be seen that the $\Delta S_{d}$ of the device for ssDNA and dsDNA reaches 2.9 × 10^8^. As illustrated in Fig. 12b, the energy bands of biomolecules with varying dielectric constants and charges reveal that the bandgap in the device is smallest for dsDNA. A continuous reduction in the bandgap is observed with the incorporation of high-k biomolecules, facilitating enhanced electron tunneling, which consequently leads to an increase in the drain current.

### The influence of $V_{ds}$, temperature ($T$) and $T_{c}$ on biosensor

**Fig. 13:**
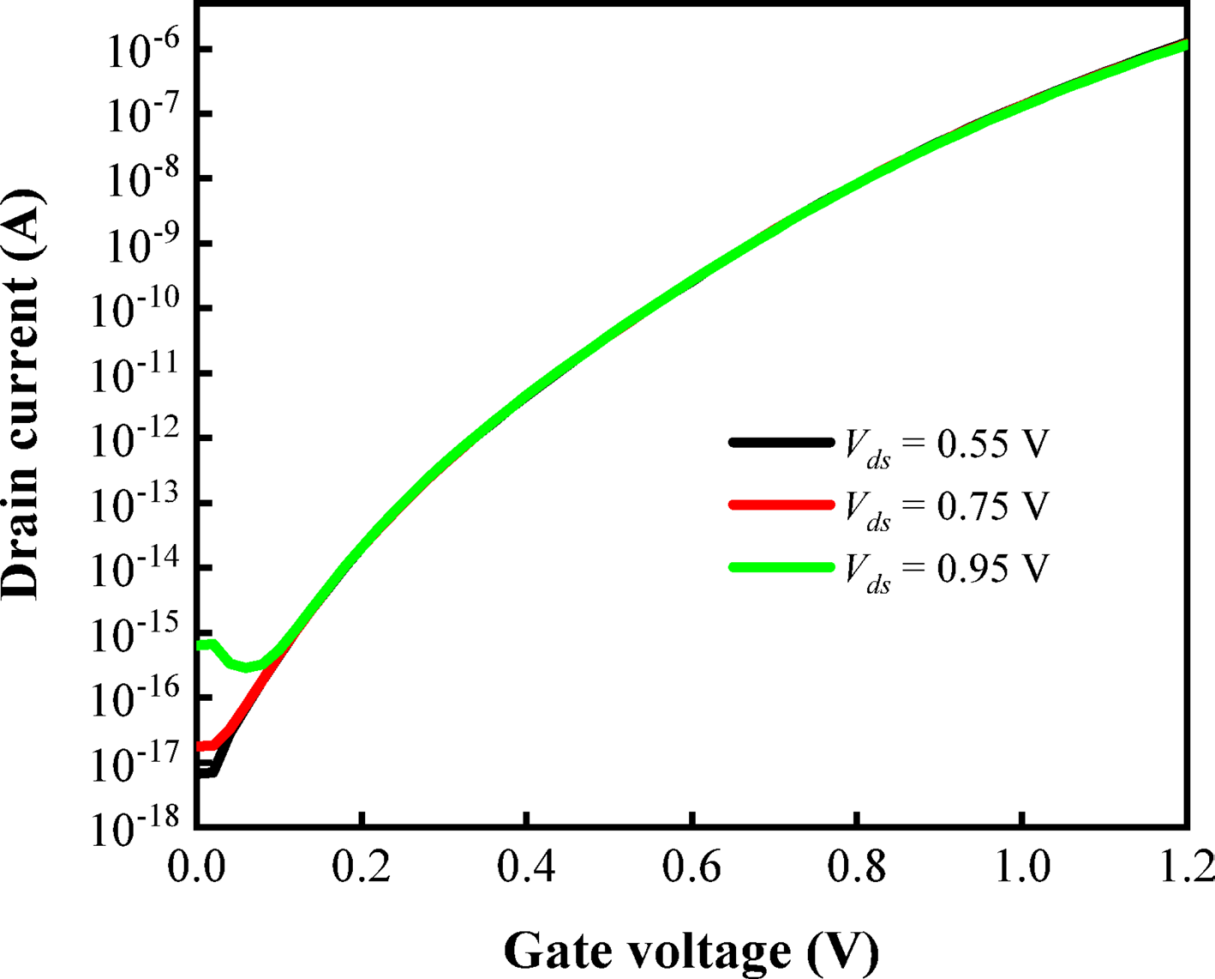
Transfer characteristics of DM-DSTG TFET based biosensor for different $V_{ds}$ at k = 12

Figure 13 illustrates the correlation between the transfer characteristic curve of the biosensor and the $V_{ds}$. As demonstrated in the figure, an increase in $V_{ds}$ results in a corresponding rise in the $I_{off}$ of the device, with the minimum value of $I_{off}$ being achieved at $V_{ds}$ = 0.55 V. Additionally, it is observed that the $I_{on}$ of the device remains relatively stable across three different values of $V_{ds}$, indicating that the device exhibits optimal performance at $V_{ds}$ = 0.55 V, where a lower switching current ratio helps reduce power consumption. In summary, the impact of $V_{ds}$ on the device performance is relatively minor within a certain range.

**Fig. 14:**
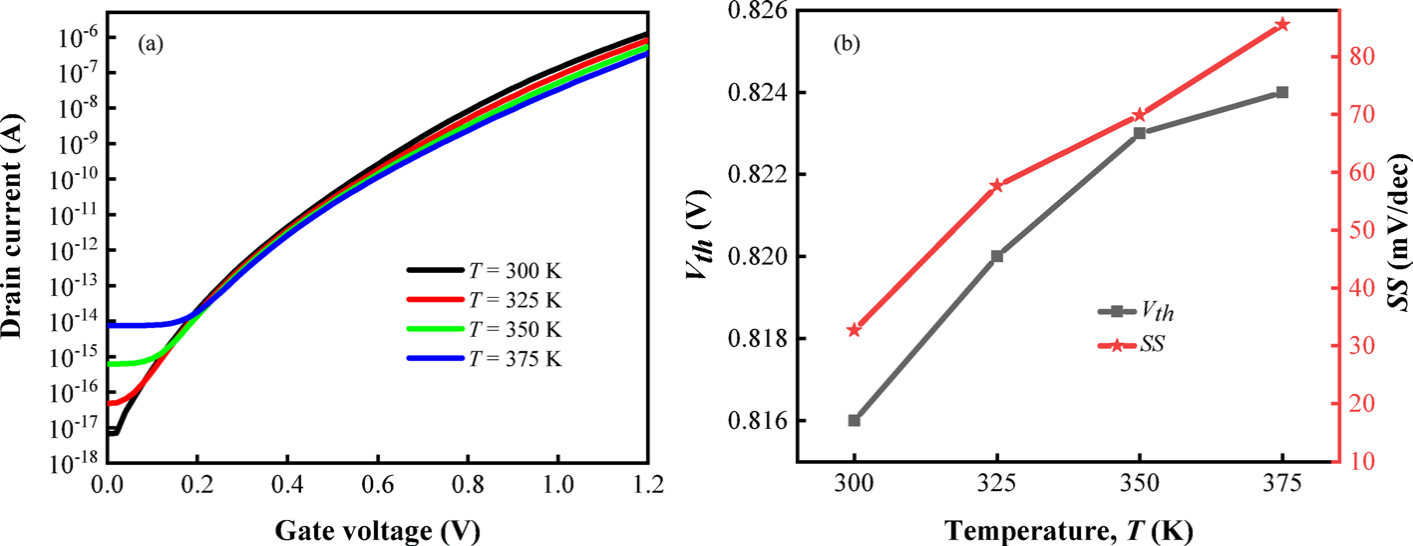
**a** Transfer characteristics, **b** $V_{th}$ and $SS$ of DM-DSTG TFET based biosensor for different $T$ at k = 12

As shown in Fig. 14a, the drain current of TFET is mainly controlled by gate and source voltages, and the change of temperature will affect the thermal excitation and tunneling probability of carriers. Therefore, with the increase of $T$, $I_{off}$ increases, and the increase of $I_{off}$ is the result of drift diffusion of a few carriers, which is greatly affected by temperature. When the $V_{gs}$ increases to a certain value, the $I_{on}$ of the device will become smaller under the influence of temperature. So it can be seen from the figure that the $I_{on}$ of the device under different temperatures changes little, the device has a higher $I_{on}$ When $T$ = 300 K. Figure 14b shows the changes of threshold voltage ($V_{th}$) and $SS$ at different temperatures. It can be seen from the figure that with the increase of $T$, $V_{th}$ and $SS$ of the device also rise, indicating that the device has a durable and stable response within this temperature range.

**Fig. 15:**
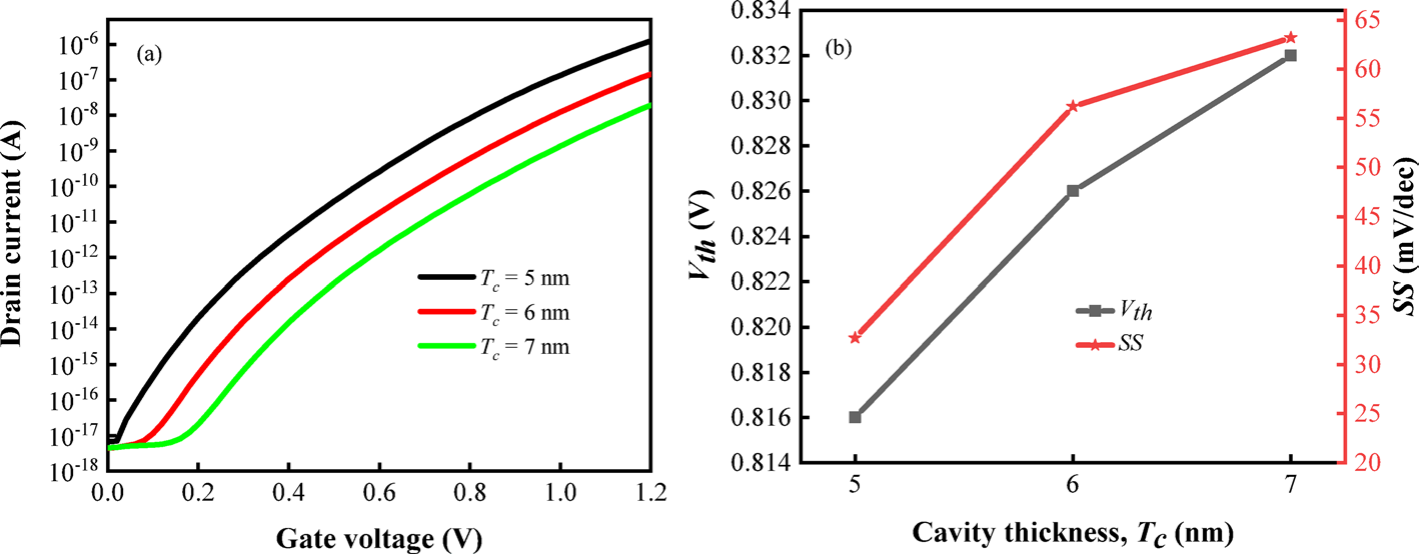
**a** Transfer characteristics, **b** $V_{th}$ and $SS$ of DM-DSTG TFET based biosensor for different $T_{c}$ at k = 12

From the preceding analysis, it is evident that the values of $S_{SS}$ and $S_{{I_{on} }}$ reach their maximum when the parameter k is fixed at 12, and thus, simulations are conducted under this condition to elucidate the influence of $T_{c}$ on the transfer characteristics of the biosensor more comprehensively. As depicted in Fig. 15a, the drain current is observed to decrease with an increase in $T_{c}$, which is attributed to an elongation of the tunneling length, resulting in a reduction in the carrier generation rate and drain current. Furthermore, as illustrated in Fig. 15b, both $V_{th}$ and $SS$ exhibit an increasing trend with the rise in $T_{c}$. The current variation of the transistor is found to be more sensitive to gate voltage fluctuations at lower $T_{c}$ values, leading to a decrease in $SS$. This result demonstrates that the enhanced performance of the DM-DSTG TFET based biosensor when the cavity thickness is minimized, as a thinner cavity facilitates greater sensitivity to biomolecular interactions.

### Comparison with existing biosensors

Numerous research efforts have been dedicated to the development of biosensors utilizing FET technology. This section aims to objectively evaluate and compare various reported FET based biosensor designs (including but not limited to TFET) with the innovative architectures proposed in this study. Table 2 provides a comprehensive comparison between the novel architecture and existing biosensors designs. Given the diverse structural and electrical characteristic parameters inherent to different biosensors, the results from various studies are tabulated. However, it is important to note that these comparisons are approximate and do not always reflect precise numerical values. The $I_{on} /I_{off}$ was taken from the maximum value of the device. Through comparative analysis, DM-DSTG TFET based biosensor achieves a higher $S_{{I_{on} }}$ and $S_{SS}$. Due to the structural optimization and material selection, it is conducive to large-scale production and reduces costs.

**Table 2:** Sensitivity comparison of the DM-DSTG TFET with other reported biosensors

References	Device structure	$I_{on} /I_{off}$	$S_{SS}$	$S_{{I_{on} }}$
Ref [11]	SiGe HDG DM TFET	10^12^	–	1.13 × 10^10^
Ref [14]	DM-DSTGTFET	1.1 × 10^10^	0.8	1.38 × 10^5^
Ref [18]	HMDG DM TFET	2.46 × 10^5^	–	10^6^
Ref [22]	DM-TMGAA-SiCFET	2 × 10^10^	0.9	–
Ref [25]	DM-SSTGTFET	2.5 × 10^9^	0.8	10^8^
Ref [28]	Si p-MOSFETs	–	0.5	3 × 10^2^
Ref [45]	GaAs GAAE-FET	–	–	9 × 10^2^
Ref [46]	SiGe HDG DM TFET	10^11^	–	1.16 × 10^10^
This work	DM-DSTG TFET	1.83 × 10^11^	0.93	2.18 × 10^10^

## Conclusions

In this study, a novel DM-DSTG TFET based biosensor is proposed, and its performance characteristics are comprehensively analyzed using the Silvaco Atlas simulator. The $I_{on}$ and $S_{{I_{on} }}$ DM-DSTG TFET based biosensor is compared with C-TFET based biosensor, demonstrating superior sensitivity in the former. The impact of various biomolecules on the device's electrical properties was systematically investigated, with particular attention given to the effects of charged biomolecules on the transfer characteristics. It is observed that positively charged biomolecules induce a more significant shift in the device's electrical parameters compared to negatively charged ones. The selectivity of the sensor is evaluated using avian influenza virus and DNA as target biomolecules, confirming its potential as a label-free biosensor. Furthermore, the influence of parameters such as $V_{ds}$ and $T$ on the device's performance is examined, revealing that the $I_{off}$ is minimally affected by $V_{ds}$, with an optimal operating temperature identified at 300 K. The device demonstrates enhanced detection capability with reduced cavity thickness, achieving optimal performance at 5 nm. The DM-DSTG TFET based biosensor achieves a remarkable sensitivity of up to 10^10^, with a $SS$ as low as 32.7 mV/decade, outperforming other TFET based biosensors. The DM-DSTG TFET based biosensor proposed in this study significantly enhances the device's sensitivity and integration in biomolecule detection through its unique parallel tunneling path design and multi-gate synergistic control mechanism.

## Data Availability

No datasets were generated or analysed during the current study.
